# Initiation of breastfeeding within 120 minutes after birth is associated with breastfeeding at four months among Japanese women: A self-administered questionnaire survey

**DOI:** 10.1186/1746-4358-3-1

**Published:** 2008-01-10

**Authors:** Yuko Nakao, Kazuhiko Moji, Sumihisa Honda, Kazuyo Oishi

**Affiliations:** 1 Department of Nursing, Nagasaki University Graduate School of Biomedical Sciences, Japan; 2 Doctoral Course of Infection Research, Nagasaki University Graduate School of Biomedical Sciences, Japan; 3 Research Center for Tropical Infectious Diseases, Institute of Tropical Medicine, Nagasaki University, Japan

## Abstract

**Background:**

The proportion of mothers in Japan who breastfeed exclusively has been low since the 1970s. The purpose of this study was to examine the association between the time of first breastfeed after birth and the proportion of mothers fully breastfeeding up to four months postpartum.

**Methods:**

A survey was conducted using a self-administered questionnaire. The participants were 318 mothers who participated in a physical examination of their four month old infants in Nagasaki City, Japan in 2003.

**Results:**

The time of first breastfeeding up to 120 minutes was significantly associated with the proportion of mothers fully breastfeeding during their stay in the clinic/hospital (p = 0.006), at one month (p = 0.004) and at four months after birth (p = 0.003). There was no significant difference in the proportion of full breastfeeding in mothers who first breastfed between the period of less 30 minutes after birth and that of between 31 and 120 minutes after birth. Logistic regression analysis indicated that the proportion of mothers who continued full breastfeeding at four months was significantly higher in those who breastfed their baby within 120 minutes compared with more than 120 minutes (OR 2.5, p = 0.01), but was not significantly different in those who breastfed within 30 minutes compared with more than 30 minutes (OR 1.8, p = 0.06). Early breastfeeding was affected by caesarean section, premature delivery and severe bleeding during delivery.

**Conclusion:**

Commencement of early breastfeeding was associated with the proportion of mothers who fully breastfed their infants up to four months. Early breastfeeding, especially within two hours, is recommended for child and maternal health.

## Background

In Japan, the proportion of mothers who breastfeed exclusively has remained low since the 1970s (Table 1) [1]. According to a national birth-cohort study involving 53,000 babies in 2001, the proportion of full breastfeeding, any breastfeeding and infant formula feeding during the first six months postpartum was 21%, 93% and 6%, respectively [2]. Another cross-sectional survey by Ministry of Health, Labour and Welfare in Japan revealed that the proportion of full breastfeeding, any breastfeeding and infant formula feeding at three months was 38%, 79% and 21%, respectively in 2005 [3]. Moreover there were only 40 baby-friendly hospitals in Japan in 2005 [4] which represents only 1.3% of the total number of Japanese maternity hospitals.

**Table 1:** Full breastfeeding at one and three months in Japan, 1960 to 2000 [1]

Year	Breastfeeding at one month (%)	Breastfeeding at three months (%)
1960	70.5	56.4
1970	31.7	31.0
1980	45.7	34.6
1990	44.1	37.5
2000	44.8	39.4

The World Health Organization (1998) summarized the findings of studies on the effects of early breastfeeding contact in Step 4 of *Evidence for the Ten Steps to Successful Breastfeeding *which states "Help mothers initiate breastfeeding within a half-hour of birth" [5,6]. However, there is a controversy about the importance of first breastfeeding within 30 minutes after delivery [7]. From observations in maternity hospitals, it is often difficult for mothers to do this, as babies may not be ready to initiate suckling [8,9]. Besides, there is evidence that caesarean section is a significant barrier to the implementation of Baby Friendly Hospital Initiative Step 4 [10]. Therefore, it is important to discuss the timing of suckling after 30 minutes.

The purpose of the present study was to determine the relationship between the time of first breastfeeding after birth and the proportion of mothers fully breastfeeding during their stay in the clinic/hospital, at one month and at four months postpartum. A survey was conducted using a self-administered questionnaire among mothers who participated in a physical examination of their four month old infants.

## Methods

A self-administered questionnaire was distributed by mail to all 391 mothers who were invited to participate in a physical examination of their four month old infants between September and December 2003 in Nagasaki, south-western Japan. Mothers were asked to answer the questionnaire voluntarily. Public health nurses collected the questionnaire on the day of the medical check-up. Three hundred and twenty-six mothers (83%) responded to the questionnaire. Among them we excluded 8 mothers who did not commence breastfeeding during their stay in the clinic/hospital. The remaining 318 participants were included in the study which was reviewed and approved by the Institutional Ethical Committee of Nagasaki University School of Medicine in August 2003.

The questionnaire included the following variables: 1) time from birth to first breastfeed, 2) method of feeding during their stay in the maternity hospital (the length of stay was about two weeks for mothers who underwent a caesarean section, and about one week for mothers who did not undergo caesarean section), and method of feeding at one month and at four months after birth, 3) characteristics of the mothers and babies (mother's age, parity, and baby's gender), 4) characteristics of delivery (gestational age, method of delivery, and amount of bleeding at delivery), and 5) characteristics of postpartum care (supplemented with sugar water or infant formula, and early skin-to-skin contact). Early skin-to-skin contact was defined as the placing of the naked baby prone on the mother's bare chest within two hours after delivery. The first breastfeed was defined as the baby suckling at the breast directly for the first time after birth.

Exclusive breastfeeding is traditionally very rare in Japan. Warm water and/or sugar water are usually given in addition to breast milk. These liquids are given from a very early stage after birth, and are frequently the first liquids given to a baby in the maternity hospital. In the present study we applied the following commonly used classification of feeding in Japan: 1) fully breastfeeding, which means that breast milk was given and infant formula was not given, regardless of whether other liquids and/or solid food were given, 2) any breastfeeding, which means including the above category 1 and that both breast milk and infant formula were given regardless of whether other liquids and/or solid food were given, and 3) infant formula feeding, which means that formula was given and breast milk was not given regardless of whether other liquids and/or solid food were given.

The time from birth to first breastfeed was classified into the following four groups: within 30 minutes, 31 to 120 minutes, 121 minutes to 24 hours, and more than 24 hours. The proportion of fully breastfeeding mothers during their stay in the clinic/hospital, at one month, and at four months, were compared between the four groups at the time of the first breastfeed. The chi-square test was used for nominal data such as baby's gender, while the Cochran-Armitage trend test was used for ordinal data such as parity. The simultaneous effects of variables on the continuation of full breastfeeding at four months were analysed by multiple linear logistic models, which included all potential risk factors (time to first breastfeeding after birth, mother's age, baby's gender, parity, gestational age, method of delivery, bleeding at delivery, early skin-to-skin contact and supplementation). The odds ratio and 95% confidence intervals were calculated for each covariate in the model. All statistical analyses were conducted using SPSS version 11.0 software (Chicago, IL, USA).

## Results

Table 2 shows the characteristics of the mothers, babies and deliveries among the 318 participants. The mean age of the mothers was 29.4 years (standard deviation 4.6 years) and 93% of infants were term. Seventeen percent of births were caesarean section. Bleeding at delivery of less than 500 ml was reported by 67% of mothers. Early skin-to-skin contact was noted to be 20%. Supplementation of sugar water or infant formula was 37% at the first feed. The proportion of mothers who breastfed within 30 minutes and within 31 to 120 minutes was 36% and 21%, respectively.

**Table 2:** Characteristics of mothers, babies and deliveries

**Characteristics**	**Number**	**%**
**Mother's age (years)**	n = 318	
18–24	43	13.5
25–29	123	38.7
30–34	106	33.3
35–41	46	14.5
**Baby's gender**		
Male	155	48.7
Female	163	51.3
**Parity**		
First	146	45.9
Second	138	43.4
Third or more	34	10.7
**Gestational age (weeks)**		
36 or less	21	6.6
37 or more	297	93.4
**Method of delivery**		
Non-caesarean section	263	82.7
Caesarean section	55	17.3
**Bleeding at delivery**		
Less than 500 ml	213	67.0
500 ml or more	85	26.7
Unknown	20	6.3
**Early skin-to-skin contact**		
Contact	64	20.1
No contact	245	77.0
Unknown	9	2.8
**Supplementation (sugar water or infant formula)**		
Supplemented	116	36.5
Non supplemented	133	41.8
Unknown	69	21.7
**Time to first breastfeed after birth**		
30 minutes or less	115	36.2
31–120 minutes	66	20.8
121 minutes to 24 hours	74	23.3
More than 24 hours	63	19.8

Table 3 shows the characteristics of the mothers, babies and deliveries and the differences in the proportion of first breastfeeding within 30 minutes and within 120 minutes. The proportion of mothers who initiated the first breastfeed within 30 minutes and within 120 minutes was not significantly different with regard to maternal age, baby's gender and parity. However, mothers who had babies with a gestational age of 36 weeks or less, had a caesarean section, or had bleeding of 500 ml or more during delivery, initiated first breastfeeding significantly later than their respective controls (within 30 minutes, p = 0.031, p < 0.001 and p < 0.001, respectively; within 120 minutes, p < 0.001, p < 0.001 and p < 0.001, respectively). Moreover, the proportion of mothers with early breastfeeding was higher in those who had early skin-to-skin contact, and in those who did not supplement feeding with sugar water or infant formula (p < 0.001).

**Table 3:** Characteristics of mothers, babies and deliveries and the proportion of first breastfeed within 30 minutes and within 120 minutes

**Characteristics**	**n**	**Within 30 minutes (%)**	**More than 30 minutes**	**p value**	**Within 120 Minutes (%)**	**More than 120 minutes**	**p value**
**Total**	318	115(36.2)	203(63.8)		181(56.9)	137(43.1)	
**Mother's age (years)**							
18–24	43	12(27.9)	31(72.1)	0.590^a^	24(55.8)	19(44.2)	0.994^a^
25–29	123	44(35.8)	79(64.2)		70(56.9)	53(43.1)	
30–34	106	40(37.7)	66(62.3)		60(56.6)	46(43.4)	
35–41	46	19(41.3)	27(58.7)		27(58.7)	19(41.3)	
**Baby's gender**							
Male	155	58(37.4)	97(62.6)	0.649^a^	90(58.1)	65(41.9)	0.687^a^
Female	163	57(35.0)	106(65.0)		91(55.8)	72(44.2)	
**Parity**							
First	146	51(34.9)	95(65.1)	0.439^b^	82(56.1)	64(43.8)	0.985^b^
Second	138	50(36.2)	88(53.8)		80(58.0)	58(42.0)	
Third or more	34	14(41.2)	20(58.8)		19(55.9)	15(44.1)	
**Gestational age (weeks)**							
36 or less	21	3(14.3)	18(85.7)	0.031^a^	4(19.1)	17(80.9)	<0.001^a^
37 or more	297	112(37.7)	185(62.3)		177(59.6)	120(40.4)	
**Method of delivery**							
Non-caesarean section	263	113(43.0)	150(57.0)	<0.001^a^	176(66.9)	87(33.1)	<0.001^a^
Caesarean section	55	2(3.6)	53(96.4)		5(9.0)	50(91.0)	
**Bleeding at delivery**							
Less than 500 ml	213	92(43.2)	121(56.8)	<0.001^c^	138(64.8)	75(35.2)	<0.001^c^
500 ml or more	85	15(17.6)	70(82.4)		31 (36.5)	54(63.5)	
Unknown	20	8(40.0)	12(60.0)		12(60.0)	8(40.0)	
**Early skin-to-skin contact**							
Contact	64	32(50.0)	32(50.0)	0.005^c^	45(70.3)	19(29.7)	0.010^c^
No contact	245	76(31.0)	169(69.0)		128(51.0)	117(47.8)	
Unknown	9	7(77.8)	2(22.2)		8(88.9)	1(11.1)	
**Supplementation (sugar water or formula)**							
Supplemented	116	25(21.6)	91(78.4)	<0.001^c^	46(39.7)	70(60.3)	<0.001^c^
Non supplemented	133	77(57.9)	56(42.1)		107(80.5)	26(19.5)	
Unknown	69	13(18.8)	56(81.2)		28(40.6)	41(59.4)	

^a ^Chi-square test; ^b ^Cochran-Armitage test; ^c ^chi-square test excluding unknown

The number of mothers who fully breastfed during their stay in the clinic/hospital was 130 (40.9%), and was 128 (40.3%) at one month, and increased to 153 (48.1%) at four months. The opposite trend was observed for "any breastfeeding". The number of mothers who carried out any breastfeeding during their stay in the clinic/hospital and at one month was 318 (100%) and 308 (96.9%), respectively, which decreased to 274 (86.2%) at four months.

**Figure 1:**
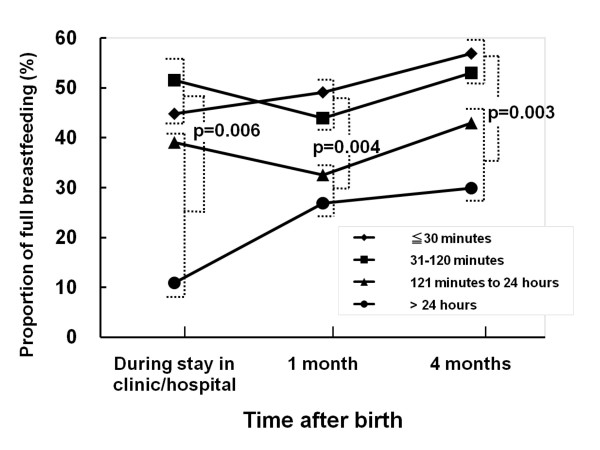
**Proportion of full breastfeeding by time after birth**. Time to first breastfeed after birth and full breastfeeding during the clinic/hospital stay, at one month and at four months. Mothers who first breastfed their baby within 120 minutes fully breastfed more than others during their stay in the clinic/hospital (p = 0.006), at one month (p = 0.004), and at four months (p = 0.003).

The proportion of fully breastfeeding was significantly associated with the time of first breastfeed (Figure 1). Mothers who first breastfed their baby within 120 minutes, fully breastfed more than others during their stay in the clinic/hospital (p = 0.006), and at one month (p = 0.004) and at four months (p = 0.003). There was a clear difference between infants first fed within 120 minutes and at more than 120 minutes. On the other hand, there was no significant difference in the proportion of mothers fully breastfeeding between the period of within 30 minutes and that of between 31 and 120 minutes at each time period (p = 0.414, 0.465 and 0.569, respectively).

In order to determine the factors influencing the continuation of full breastfeeding, a multiple linear logistic model analysis was conducted. Table 4 shows the estimate and the 95% confidence interval of the odds ratio (OR) with its *p *value for each factor included in the model. The proportion of mothers who continued full breastfeeding at 4 months was 1.8-fold higher in those who breastfed within 30 minutes than in those who breastfed at more than 30 minutes, but the odds ratio was not statistically significant (p = 0.061). The proportion of mothers who continued full breastfeeding at four months was significantly higher in those who breastfed their baby within 120 minutes (OR 2.5, p = 0.01) compared with those who breastfed at more than 120 minutes. The odds ratio for those who breastfed within 30 minutes (OR 1.8) and that for those who breastfed within 120 minutes (OR 2.5) were not statistically different. Furthermore, the proportion of mothers who continued full breastfeeding at four months was significantly higher of about 2-fold in female infants than in male infants.

**Table 4:** Logistic regression analysis: Variables influencing full breastfeeding at 4 months

**Factor**	**Comparison**	**Odds Ratio**	**95%CI**	**p value**
**Model 1 (≤ 30 minutes vs > 30 minutes)**				
Time to first breastfeed	≤ 30 minutes vs > 30 minutes	1.82	0.97, 3.43	0.06
Mother's age	≥ 30 years vs < 30 years	0.62	0.34, 1.11	0.11
Baby's gender	Female vs male	2.16	1.24, 3.77	<0.01
Parity	Multiparous vs primiparous	1.36	0.76, 2.43	0.40
Gestational age	≥ 37 weeks vs ≤ 36 weeks	1.36	0.36, 5.24	0.65
Method of delivery	Non Caesarean section vs Caesarean section	1.29	0.53, 3.12	0.57
Bleeding at delivery	< 500 ml vs ≥ 500 ml	1.29	0.62, 2.67	0.50
Early skin-to-skin contact	Non contact vs contact	0.92	0.46, 1.86	0.82
Supplementation	Non supplemented vs supplemented	1.62	0.91, 2.91	0.10

**Model 2 (≤ 120 minutes vs > 120 minutes)**				
Time to first breastfeed	≤ 120 minutes vs > 120 minutes	2.45	1.21, 4.95	0.01
Mother's age	≥ 30 years vs < 30 years	0.64	0.35, 1.15	0.14
Baby's gender	Female vs male	2.15	1.23, 3.76	<0.01
Parity	Multiparous vs primiparous	1.39	0.77, 2.49	0.28
Gestational age	≥ 37 weeks vs ≤ 36 weeks or less	1.07	0.27, 4.25	0.92
Method of delivery	Non Caesarean section vs Caesarean section	1.02	0.41, 2.59	0.96
Bleeding at delivery	< 500 ml vs ≥ 500 ml	1.29	0.62, 2.69	0.49
Early skin-to-skin contact	Non contact vs contact	0.90	0.45, 1.80	0.76
Supplementation	Non supplemented vs supplemented	1.45	0.80, 2.65	0.23

CI = confidence interval

## Discussion

There are a number of limitations of this study. Firstly, since the study design was retrospective, some recall bias in mothers relating to the time to first breastfeed may have affected our results. Secondly, as the participants took part in a physical examination of their four month old infants, some selection bias or confounding factors may have occurred. Among 391 mothers who were mailed to a questionnaire, some mothers did not participate in a physical examination of their infants because of their baby's or their own health problem. Therefore, the study participants and their baby could be healthier than those who did not participate in the present study. However, since the response was high (83%), the extent of selection bias may be small. Another possible source of bias was that mothers who put the baby to their breast in the first two hours were more motivated to breastfeed than the other mothers. Their infant feeding intention could lead to continuation of fully breastfeeding their infants up to four months. As it is not feasible to conduct a randomized controlled trial on this topic, we thought that the information collected in the study was worth analysing.

The present study revealed four findings associated with initiation and maintenance of breastfeeding in Japanese women. Firstly, 36% of 318 mothers breastfed within 30 minutes, and 57% breastfed within 120 minutes. If more women could be encouraged to start breastfeeding early we may see a higher rate of full breastfeeding. Secondly, severe bleeding during delivery, premature delivery, and caesarean section were obstacles for early breastfeeding. If it is possible to attempt early breastfeeding during these conditions, for example, by delaying routine neonatal procedures and keeping mother and baby in close proximity [10], we can further promote early breastfeeding. Thirdly, mothers who initiated breastfeeding within 120 minutes maintained full breastfeeding during their stay in the clinic/hospital, at one month and at four months after birth. The rate of decline in breastfeeding was significantly slower for infants who were first suckled early than for those who were first suckled later [11,12]. Fourthly, the proportion of full breastfeeding mothers did not differ between first breastfeeding within 30 minutes and first breastfeeding within 31 to 120 minutes. Righard reported that infants require 49 minutes on average in order to initiate breastfeeding through their own efforts and sucked for about 20 minutes [9]. Widstrom reported that it takes 55 minutes for infants to start suckling after birth [8]. On the other hand, catecholamine is secreted rapidly within 120 minutes by newborn infants, this period is called "the newborn infant awaking term" [13]. A previous study revealed that first breastfeeding within 120 minutes was significantly associated with skin-to-skin contact, and extended the duration of subsequent breastfeeding [14]. We should not hurry to commence breastfeeding immediately after delivery, as the first breastfeed within 120 minutes was associated with maintenance of breastfeeding up to four months. It might be suggested that the recommended timing of the first breastfeed should be extended to within 120 minutes.

In the present study, the proportion of mothers who continued full breastfeeding at 4 months was higher in female infants than in male infants. A previous study [15] in Latin American countries (Brazil, Honduras and Mexico) reported that male infant was significantly associated with cessation of exclusive breastfeeding. Another study [16] in Greece analyzed factors affecting intention to breastfeed, and found that male gender was associated with negative attitude of breastfeeding. It is possible that mothers may think that male infants need more nutrition than female infants, and they may add infant formula earlier. To the contrary, a study in China [17] reported that girls were breastfed for a significantly shorter period than boys because of preference for sons. Another possible reason for the lower proportion of full breastfeeding in male infants was that male infants might have more medical problems and more difficulties with breastfeeding. A study [18] suggested that infants with ankyloglossia, which is more common in male than in female, had more difficulties with breastfeeding, defined as nipple pain lasting longer than 6 weeks and/or difficulty of the baby latching onto the breast. The higher frequency of medical problems in male infants might have affected the lower proportion of full breastfeeding at four months.

The mother who has early contact with her baby and initiates breastfeeding after delivery might have enhanced maternal behaviour compared with the mother who does not [19]. Early contact with the baby immediately after birth will promotes a closer relationship between a mother and her baby. Moreover, the early initiation of breastfeeding gives the mother a strong sense of satisfaction [20]. Maternal satisfaction with first breastfeeding was associated with early initiation of breastfeeding within 120 minutes in our study (data not shown). Moreover, some mothers responded to our questionnaire, and described their feelings about breastfeeding as "I felt motherly love", "I increased the pleasure of childbirth" and "I felt calm". Early breastfeeding helped to calm mothers and make them feel relaxed. Early breastfeeding not only increased the proportion of mothers maintaining full breastfeeding, but produced positive mental effects in the mothers.

## Conclusion

Commencement of breastfeeding within two hours after delivery was associated with the continuation of full breastfeeding at four months. The observed association between early initiation of breastfeeding and maintenance of full breastfeeding should be confirmed by a prospective cohort study, in which some selection bias and recall bias can be eliminated. Helping mothers initiate early breastfeeding, especially within two hours, is strongly recommended for child and maternal health.

## Competing interests

The author(s) declare that they have no competing interests.

## Authors' contributions

YN: project design, date collection, data analysis and paper writing. KM: data analysis and paper writing. SH: data analysis and paper writing. KO: project design.
